# How bifurcations affect functional connectivity in finite-size neural networks

**DOI:** 10.1186/1471-2202-16-S1-P193

**Published:** 2015-12-18

**Authors:** Anna Cattani, Diego Fasoli, Stefano Panzeri

**Affiliations:** 1 Neural Computation Laboratory, Center for Neuroscience and Cognitive Systems@UniTn, Istituto Italiano di Tecnologia, Rovereto, Italy

## 

Understanding how the functional connectivity of a neural network (i.e. the statistical dependencies among different neurons) depends upon its anatomical connectivity and how it is modulated by other network parameters is a central topic in neuroscience [1]. However, only few analytical investigations of functional connectivity in network models have been reported [2]. A difficulty for such analytical studies is that they require in principle characterizing the bifurcation points (i.e. the parameter values at which the network undergoes a sudden change in dynamics) and how functional connectivity changes close to such points. Given that biological networks have a finite number of neurons, it is also important to develop such analytical studies without relying only on large network approximations that neglect finite-size effects. To overcome these difficulties, here we developed a new approach to investigate the dynamics of finite-size networks composed by interconnected inhibitory and excitatory neurons. We applied this approach to a network of neurons described by voltage-based differential equations, with an input to each neuron provided by a deterministic current superimposed to a weak normally distributed stochastic component.

To understand the interplay between the deterministic dynamics of the network and the effects of random fluctuations in a finite-size network, we proceeded in two steps.

First, we neglected the stochastic component of the input and we performed a complete numerical and analytical bifurcation analysis while varying the deterministic inputs and the strength of the connections. We did this using the *MatCont *continuation package [3] and the analytical expressions of the Jacobian matrix eigenvalues. We found that our finite-size network displayed standard bifurcations such as saddle-node and Hopf manifolds. Specifically, these manifolds describe the input currents values for which equilibria collide and annihilate to each other, and for which self-sustained oscillations arise, respectively. Moreover, strong inhibition gave rise to additional branch point bifurcations: for highly negative synaptic weights, the system exhibited special multiple solutions characterized by heterogeneous membrane potentials. These particular solutions represent new finite-size effects not captured by large network approximations.

Second, we inserted stochastic components into the input and studied the behaviour of the network under the effect of noise. Specifically, we used a new first-order perturbative method where the perturbative parameter is the standard deviation of the noise in the input. This technique allowed the analytical calculation of the cross-correlation between firing rates, and of the Fisher information about the external input carried by the network. Our analytical solutions showed that the neurons become independent when the deterministic component of the input is strong, while they become correlated for smaller current values lying on the saddle-node manifold. The latter is due to a counter-intuitive finite-size effect [4] that can be fully captured by our formalism. Furthermore, in our network strong correlations led to large Fisher information values, thus enhancing the encoding precision of the network with respect to the input variables. This also shows that reducing correlations among neurons activity does not imply an enhancement of the information encoding, as suggested in some studies [5].

## References

[B1] Buice MA Chow CC Dynamic Finite Size Effects in Spiking Neural Networks PLoS Computational Biology 2013 9 1 e100287 10.1371/journal.pcbi.1002872 PMC3554590 23359258

[B2] Pernice V Staude B Cardanobile S Rotter S Recurrent Interactions in Spiking Networks with Arbitrary Topology Physical Review E 2012 85 031916 10.1103/PhysRevE.85.031916 22587132

[B3] Dhooge A Govaerts W Kuznetsov YA Matcont: A matlab package for numerical bifurcation analysis of ODEs ACM Trans Math Softw 2003 29 2 141 164

[B4] Steyn-Ross ML Steyn-Ross DA Sleigh JW Modelling general anaesthesia as a first-order phase transition in the cortex Progress in Biophysics & Molecular Biology 2004 85 2-3 369 385 15142753 10.1016/j.pbiomolbio.2004.02.001

[B5] Ecker AS Berens P Keliris GA Bethge M Logothetis NK Tolias AS Decorrelated Neuronal Firing in Cortical Microcircuits Science 2010 327 5965 584 587 20110506 10.1126/science.1179867

